# Tocopherol induced angiogenesis in placental vascular network in late pregnant ewes

**DOI:** 10.1186/1477-7827-8-86

**Published:** 2010-07-12

**Authors:** Ramanathan K Kasimanickam, Vanmathy R Kasimanickam, Jacobo S Rodriguez, Kevin D Pelzer, Philip D Sponenberg, Craig D Thatcher

**Affiliations:** 1 College of Veterinary Medicine, Washington State University, Pullman, WA 99164, USA; 2 Virginia-Maryland Regional College of Veterinary Medicine, Virginia Polytechnic Institute and State University, Blacksburg, VA 24061, USA; 3 College of Nursing and Health Innovation, Arizona State University, Phoenix, AZ 85004, USA

## Abstract

**Background:**

Tocopherols have biphasic, proangiogenic and antiangiogenic therapeutic effects. The objective of this clinical trial was to clarify tocopherol's placental angiogenic potential in late pregnant ewes following oral supplementation.

**Methods:**

Eighteen pregnant ewes during late gestation were selected for this study. Ewes were given oral supplementation of 500 mg of alpha-tocopherol (aT; N = 6) or 1000 mg of gamma-tocopherol (gT; N = 7) or placebo (CON; N = 5) once daily from 107 to 137 days post breeding. Serum was obtained at weekly intervals and tissue samples were obtained at the end of supplementation to: 1) evaluate tocopherol concentrations in serum, uterus and placentome; 2) evaluate relative mRNA expressions of Vascular Endothelial Growth Factor (VEGF), Placental Growth Factor (PlGF), endothelial Nitric Oxide Synthase (eNOS) and Hypoxia Inducible Factors (HIF) in uterus, caruncle and cotyledon; 3) analyze the morphometry of the placental vascular network.

**Results:**

Supplementation of aT or gT resulted in increased concentrations in serum, placentome and uterus compared to control (P < 0.05). In aT group, mRNA expressions of PlGF, eNOS and HIF-1α in cotyledon were greater than the CON group. In gT group, mRNA expressions of VEGF, eNOS, HIF-1 alpha and HIF-2 alpha in caruncle and uterus, and HIF-1α in cotyledon, were greater than the CON group. Morphometry analysis revealed increased angiogenesis in the supplemented groups.

**Conclusion:**

Daily oral supplementation of aT or gT increased angiogenesis in the placental vascular network in pregnant ewes during late gestation. Increase in placental angiogenesis may provide nutrients required for the development and growth of fetus during late pregnancy.

## Background

Angiogenesis is the process of development of new blood vessels from an existing vascular network [1]. Hypoxia constitutes a basic regulatory mechanism in vascular development during embryonic and fetal growth *in utero *[2]. In endothelial cells, hypoxic conditions drive the transcription of multiple genes which control vascular function, expansion and remodeling. Although tissue hypoxia is the main driving force for angiogenesis, a growing body of evidence has demonstrated that oxidative stress can also be a potent trigger for the development of new vessels [3-6].

Two families of radicals, reactive oxygen species (ROS) and reactive nitrogen species (RNS) are very crucial to growth, differentiation, apoptosis, and aging. The transition from growth to degeneration of cells is finely tuned by the relative concentration of oxidants. For instance, in ambient conditions, H_2_O_2 _in nano-micromolar concentrations are necessary for angiogenesis [7], while its excessive accumulation at levels more than 150-200 μM prompts endothelial damage [8]. An imbalance of free radicals attacks and alters cell constituents, resulting in lipid peroxidation, protein peroxidation, oxidative inactivation, mutation of DNA, and destruction of vitamins and other functions that protect cell components [9]. Excessive accumulation of free radicals affects placental development and function, and may subsequently impact both the fetus and dam [10-13]. The dietary antioxidants are important in attenuating the oxidative damage produced by radicals [13,14]. Tocopherols are micronutrients which act as free radical scavengers. The major tocopherols found in mammalian tissue are alpha-tocopherol (aT) and gamma-tocopherol (gT). Studies on tocopherol in humans have shown that aT constitutes 80 to 90% of vitamin E in plasma, which is due to the preferential incorporation of aT into very low-density lipoprotein via tocopherol-binding protein, and a rapid catabolism of gT within the liver [14]. Consequently, aT has received the most attention and is the primary form of vitamin E in supplements. However a recent work has suggested that under specific circumstances gT might scavenge reactive species more effectively than aT thereby, being more beneficial [15,16]. Gamma tocopherol affords higher protection against lipid peroxidation as compared to aT [17]. It has been demonstrated that high levels of aT intake can decrease gT [17]. Also gT has synergistic effect with other tocopherols and suggested as most promising agent to use. Hence we decided to use both tocopherols in this study. Human and animal studies have demonstrated that chronic supplementations with tocopherols have biphasic, proangiogenic and antiangiogenic therapeutic effects [18-22]. A recent study also concluded that tocoretinols not tocopherols suppressed Vascular Endothelial Growth Factor (VEGF) induced angiogenesis [23].

Optimal placental development is critical for fetal development and growth. Normal fetal growth is genetically pre-programmed and regulated by fetal, placental, maternal and environmental factors. A range of pathophysiological factors including maternal stress due to poor nutrition, hyperthermia or metabolic diseases such as pregnancy toxemia and eclampsia may affect important metabolic, transport and hemodynamic functions of placentas. This may lead to fetal stress due to poor supply of nutrition and thus impairment in fetal growth. A recent study demonstrated that glucocorticoid induced restriction of fetal and placental growth is mediated through reduced placental expression of VEGF and associated reduction in placental vascualrization during late pregnancy [24]. Supplementation of antioxidants may negate oxidative stress, promote angiogenesis and increase nutrient supply to the fetus. The differential expression of VEGF and placental growth factors (PlGF) throughout gestation indicates that they both play essential roles in placental angiogenesis. Hypoxia inducible factor (HIF)-1α or HIF-2α combine with HIF-1β to form the functional basic helix-loop-helix transcription factor HIF-1 to stimulate transcription of the VEGF gene via interaction with the hypoxia response element (HRE) [25]. Several genes encoding growth factors and tyrosine kinase receptors essential for endothelial cell growth and function such as erythropoietin (EPO), VEGF, endothelial Nitric Oxide Synthase (eNOS) and Flk-1 were shown to contain HIF-binding sites in their promoter/enhancer sequences.

We hypothesize that oral supplementation of tocopherols during late gestation in ewes will increase tocopherol levels in the circulation, resulting in increased concentrations in the uterus and placenta and thereby increasing the expression of angiogenic genes and exerting an angiogenic effect. The objectives of the study were to: 1) compare aT and gT concentrations in serum, uterine and placentomal tissues among supplemented and CON groups; 2) compare angiogenic gene (VEGF, PlGF, eNOS, and HIFs) expressions in the uterus, caruncle and cotyledon among supplemented and CON groups; and 3) compare angiogenic morphometric parameters of the uteroplacental vascular network of late pregnant ewes supplemented with aT, gT, or placebo.

## Methods

Eighteen pregnant ewes (2 to 6 years of age and weighing approximately 68 kg; impregnated by different sires by natural service), with similar breeding dates were selected for the current study and were mainatined under normal pasture conditions. One week prior to the trial, the selected ewes were moved to the research facility and were penned by treatment groups. The ewes had access to 35 sq ft/ewe paddock lots. In addtion they were fed 250 g of concentrate/ewe/day and free choice hay. The composition of hay is given in Additional file 1, Table S1. This study was approved by Virginia Tech institutional animal care and use committee (IACUC; 04-068-CVM). Tissue Use Protocol was approved by IACUC at Washington State University (ASAF #03922-001).

**Figure 1:**
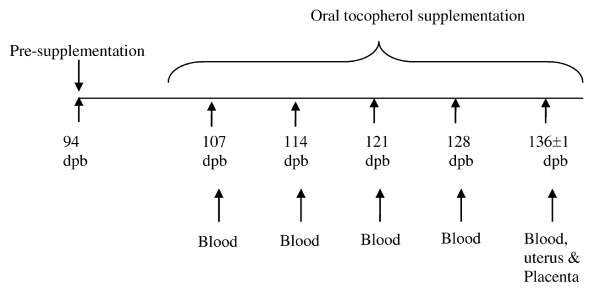
**Schematic representation of treatment regimen and sample collection**. dpb - days post breeding.

The ewes were randomly assigned to three groups: 1) aT group (n = 6) - received 500 mg of aT (Nature's Bounty, Bohemia, NY 11716), 2) gT group (n = 7) - received 1000 mg of gT (Kemin Industries Inc., Des Moines, Iowa 50317), 3) Control (CON) group (n = 5) - received a placebo. The average tocopherols concentrations in the supplement is given in Additional file 2, Table S2. Animals were supplemented orally, once daily, from approximately 100 to 137 days post breeding (dpb) (Figure 1). Blood samples were collected one week prior to onset of supplementation and then weekly until the end of the supplementation period [94 dpb (presupplement level), 107 dpb, 114 dpb, 121 dpb, 128 dpb, 136 ± 1 dpb] for serum aT and gT evaluation (Figure 1). At the end of the supplementation period (136 ± 1), all ewes were euthanized and tissue samples were collected from the gravid uterus and placentomes to evaluate aT and gT concentrations. Tissue samples were snap frozen and stored at -70°C until analysis. Caruncle, cotyledon and intercaruncular uterus samples were also collected and stored in RNAlater (Qiagen Inc., Valencia, CA 91355) and frozen at -70°C to evaluate mRNA expression. Cotyledones were separated from caruncles by applying strong pressure. Placentomal and uterine tissues were collected close to the umbilical cord for consistency.

### Serum tocopherol estimation

The High Performance Liquid Chromatography (HPLC) method described by Panfili et al (2003) was employed for the quantification of tocopherols [26]. For each sample, dose-dependent (10, 50, 100, 500 and 1000 mg/L) tocopherols (Sigma-Aldrich, St. Louis, MO 63103) positive control were prepared. In a glass tube, 1 mL of serum sample and 20 μL of aT acetate (1000 mg/L) in ethanol (internal standard to estimate yield of extraction) was added and vortexed. To this 1 ml of ethanol containing 0.01% of butylated hydroxytoluene (BHT) was added. The tocopherols were extracted 3 times with 1 ml of cyclohexane containing 0.01% BHT. After evaporation of the pooled cyclohexane fractions under a stream of nitrogen, the residues were reconstituted in 200 μL of cyclohexane. The samples were filtered through a Pall Gellman Acrodisc^® ^(0.2 μm/13 mm) into a HPLC vial and subjected to a HPLC analysis to quantify the tocopherol's concentration.

### Uterine and placentomal tissue tocopherol estimation

The HPLC method described by Katsanidis and Addis (1999) was employed for quantification of tocopherol concentrations in uterus and placentome samples [27]. Each uterine and placentomal tissue sample was homogenized separately in water (1:2) containing ascorbic acid (62.5 g of ascorbic acid in 250 mL of double distilled water) to protect tocopherol from air oxidation during saponification process [27]. The tissue homogenates were aliquoted and frozen and stored at -70°C until analysis. One point five grams of uterus or placental homogenate was placed in a screw cap tube and 0.125 g of ascorbic acid and 3.5 mL of the saponification solution [55% ethanol in distilled water and 11% KOH (w/v)] were added to the tissue. The tube with sample was heated to 80°C for 15 min. After saponification, the tubes were cooled under tap water for 1 min, and then 1 mL of distilled water was added. The solutions were extracted 3 times with 1 mL of cyclohexane containing 0.01% of BHT. The 3 extracts were combined and the solvent was evaporated under reduced pressure by a rotovap. The residue was reconstituted in 500 mL of ethanol containing 0.01% BHT.

An Agilent Technologies 1100 series liquid chromatography system (Agilent Technologies Inc. Santa Clara, CA 95051, USA) was used to perform HPLC analysis to quantify tocopherols in supplement, serum, and tissue samples. The analysis was conducted in normal phase, with a HPLC column Agilent Technologies Zorbax Rx-Si 150 × 4.6 mm, 5 μ with an elution solvent constituted of hexane-isopropanol (99.7:0.3) with a flow rate of 1 mL/min and an injection volume of 10 μL. The tocopherols were analyzed by fluorescence with an excitation wavelength of 295 nm and emission wavelength of 330 nm.

### Real Time Polymerase Chain Reaction

Total RNA was extracted with RNeasy Mini Kit (QIAGEN Inc. Valencia, CA, USA) according to the manufacturer's protocol from uterine and placental tissues. Total RNA (1 μg) was reverse transcribed into cDNA with a SuperScript II reverse transcriptase (Invitrogen, Carlsbad, CA 92008, USA) using random hexamers as a primer. Primers for the genes examined are listed in Table 1. To ensure the amplification of a single amplicon of the expected size, the product was viewed on an ethidium bromide stained electrophoresis gel. The RT-PCR analyses for VEGF, PlGF, eNOS, HIFs and 18 S rRNA were performed using the ABI 7300 Sequence Detection System instrument and software (PE Applied Biosystems Inc., Foster City, CA, USA). To compare mRNA expression levels among samples, mRNA for each gene of interest was normalized to the expression of a housekeeping gene, 18 s RNA, and differences in gene expression were calculated as a fold change to the mean of the vehicle group using the 2^-ΔΔCt ^method [28,29].

**Table 1:** Primer sequences for Vascular Endothelial Growth Factor, Placental Growth factor, endothelial Nitric oxide Synthase and Hypoxia Inducible Factors

Gene	Primer	Sequence 5' to 3'	GenBank accession number
VEGF	Forward	TGTAATGACGAAAGTCTGGAG	AF071015
	Reverse	TCACCGCCTCGGCTTGTCACA	
PlGF	Forward	TGCCGGTCATGAGGCTGT	NM_002632*
	Reverse	GCAGTCACTGAAGAGTGTGAC	
eNOS	Forward	TGGGCCGCATCCAGTG	NM_001129901
	Reverse	GAACATCTCCTGTGCTGAGCTG	
HIF-1α	Forward	TCAGCTATTTGCTGTGAGG	EU340260
	Reverse	TTCACAAATCAGCACCAAGC	
HIF-1β	Forward	AGA TGC AGG AAT GGA CTT GG	EU340261
	Reverse	CCT GGC CTT TTA ACT TCA CG	
HIF-2α	Forward	AAGTCAGCCACCTGGAAGG	EU340264
	Reverse	TCACACACATCATGCACTGG	
HIF-2β	Forward	AGGATGAGGTGTGGAAATGC	EU340265
	Reverse	CCTCAGAGTGGCAGAACTCC	

*Homology to human

### Morphometry analysis

**Figure 2:**
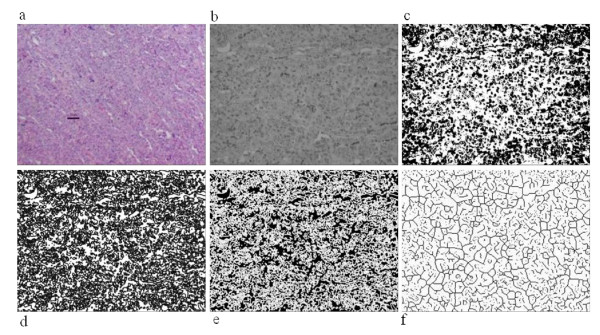
**Steps involved in image processing of histological sections of placentome using ImageJ 1.42 q**. a. histological image of placental vascular network (Bar = 50 μm; 100× magnification); b. 8 bit image; c. Image smoothened; d. Image with edges; e. Binary conversion; f. Skeletonization.

The placentome samples collected for histological evaluation were fixed in 10% neutral buffered formalin, sectioned at 5 microns, and stained with hematoxylin and eosin. They were evaluated on a Nikon E400 Eclipse microscope, and photomicrographs (100×) were taken with a Nikon camera with a 3 chip. Images were processed with Nikon Act 1 software. Image processing and morphometry analysis were performed using ImageJ 1.42 q (NIH, USA) to evaluate the fractal dimension and lacunarity [30,31]. The main processing steps applied to the images are shown in Figure 2. Briefly, after downloading the original image in the software, they were converted to 8 bit black and white images, and thresholding was performed by selecting mean value as threshold value. The images were made binary and were skeletonized.

**Figure 3:**
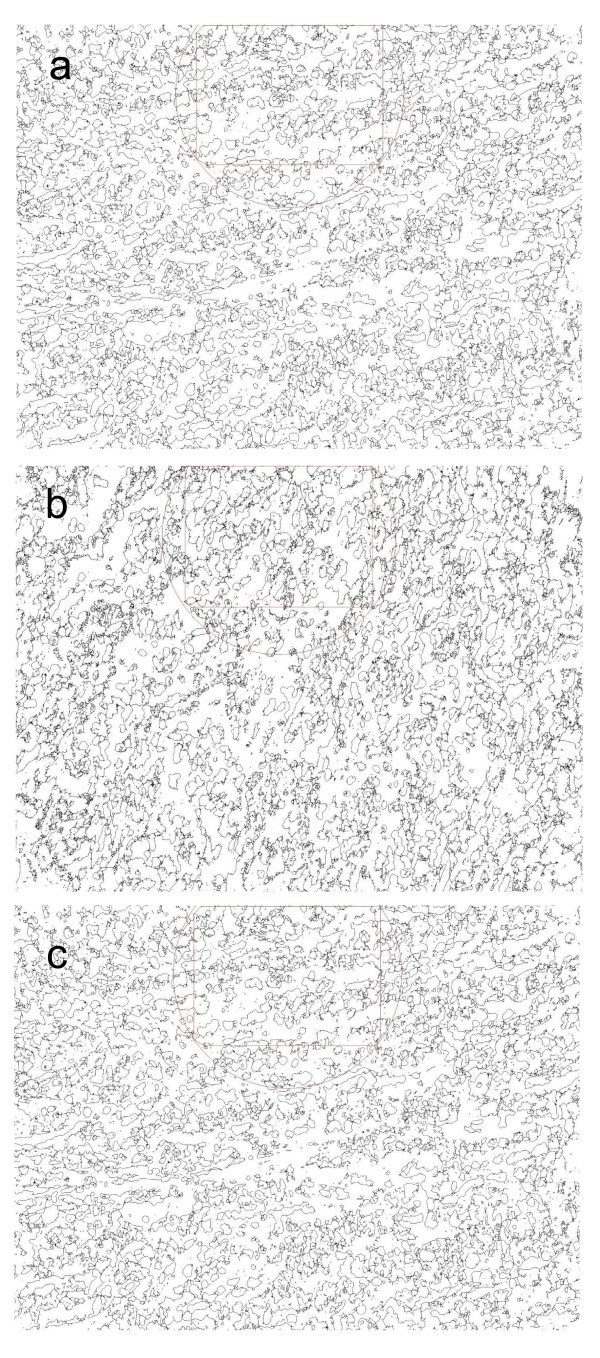
**Morphometric analysis of placental vascular network of late pregnant ewes supplemented with alpha tocopherol, gamma tocopherol or placebo**. a. Morphometric parameter of placental network of ewe supplemented with γ tocopherol. Field demarked as red-square had Fractal dimension 1.6204 and lacunarity 0.4967. A total of 30 fields were evaluated for each sample; b. Morphometric parameter of placental network of ewe supplemented with α tocopherol. Field demarked as red-square had Fractal dimension 1.7030 and lacunarity 0.5884. A total of 30 fields were evaluated for each sample. c. Morphometric parameter of placental network of ewe treated with placebo. Field demarked as red-square had Fractal dimension 1.6263 and lacunarity 0.6033. A total of 30 locations were evaluated for each sample.

Fractal analysis is a contemporary method of applying nontraditional mathematics to patterns that defy understanding with traditional Euclidean concepts. Fractal analysis measures complexity using the fractal dimension. A fractal dimension is, in essence, a scaling rule comparing how a pattern's detail changes with the scale at which it is considered. The fractal dimension is a valuable parameter to describe the complexity. FarcLac 2.5 release 1b5 m (NIH, USA) was used to perform fractal dimension. The FracLac scan images using a shifting grid algorithm that can do multiple scans from different locations on each image. The fractal dimension of the binary skeleton was estimated using the box counting method at multiple origins. The basic procedure is to systematically lay a series of grids of decreasing calibre (the boxes) over an image and record data (the counting) for each successive calibre. To minimize grid location effects, the algorithm started from number of locations, generating a set of variables for fractal dimension. The average value over all locations was considered as the final estimate of fractal dimension. During the same analytical process 'Lacunarity" was also calculated. This parameter is a measure of homogeneity of structure or the degree of structural variance within an object. It was estimated as the average of the coefficient of variation for pixel density over all grid sizes and locations. The field demarked as a square in Figure 3 is one location. A total of 30 locations were evaluated for each sample.

### Statistical analyses

The MIXED procedure of the SAS System (SAS version 9.12, SAS Institute Inc., Cary, NC 27513) was used to perform a repeated measures analysis of covariance to test for effects of treatments (aT, gT and CON), days post breeding [94 dpb (pre-supplement level), 107 dpb, 114 dpb, 121 dpb, 128 dpb), 136 ± 1 dpb] and their interactions on the serum concentration of tocopherols. Covariation of repeated measurements was modeled as compound symmetric and model adequacy was assessed using plots of standardized residuals. The differences in the serum tocopherol concentrations between pre-supplement stage and post supplement stages of gestation were tested by creating contrast statements. Analysis of variance was used to determine the within treatment between days post breeding differences and within days post breeding between treatment differences. Since the data were not normally distributed, a non-parametric procedure was used to perform the Kruskal-Wallis method test to compare the median tocopherol concentrations in serum, placenta and uterus among the treatment groups (Table 2). For responses with significant differences, pair-wise comparisons were performed using Minitab 12.1 (Minitab Inc., State College, PA, USA).

**Table 2:** Median (25^th ^and 75^th ^percentile) tissue concentrations of alpha- and gamma-tocopherols between the treatment groups in ewes (N = 18)

		Concentrations	
Tissue	Treatment Groups	aT (mg/kg)	gT (μg/kg)
Placenta	aT	9.19 (6.77, 12.1)^a^	45.5 (10.0, 83.3)^a^
	gT	2.74 (2.26, 6.22)^b^	607 (477, 1057)^b^
	Control	2.88 (1.85, 4.16)^b^	92.4 (57.6, 153.0)^a^
Uterus	aT	5.23 (4.38, 6.50)^a^	0 (0, 189.3)^a^
	gT	3.28 (2.34, 3.54)^b^	737.7 (607.1, 979.6)^b^
	Control	2.34 (1.91, 3.87)^b^	156.5 (110.3, 237.1)^c^

^abc^different superscripts between treatment groups within tissue are significantly different (P < 0.05); aT- alpha tocopherol; gT-gamma tocopherol; CON - Control

The RT-PCR data were subjected to ANOVA using 2^-ΔΔCt ^values to ascertain statistical significance of any differences in VEGF, PlGF, eNOS and HIFs expression in placebo vs. tocopherol-treated groups [29]. The differences in fractal dimension and lacunarity among treatment groups were tested by non-parameteric Kruskal-Wallis method. There were 3 ewes (2 in CON and 1 in aT) that had twins. The the results did not change after inclusion, exclusion averaging the the values from twins; however excluded from the analysis.

## Results

### Serum tocopherol concentrations

Serum tocopherol concentrations were significantly different between treatment groups (P < 0.0001) and days post breeding (P < 0.0001; Additional file 3, Table S3). Significant treatment × days post breeding interaction was observed (P < 0.0001; Additional File 3, Table S3). The pre-supplementation serum levels of aT compared to post-supplemental stages of gestation were different between aT and gT groups (P < 0.0001; Additional file 4, Table S4), and between aT and CON groups (P < 0.0001; Additional file 4, Table S4) and not between gT and CON groups (P > 0.05; Additional file 4, Table S4). The pre-supplementation gT serum concentrations compared to other stages of gestation were different between gT and aT groups (P < 0.0001; Additional file 4, Table S4), and between gT and CON groups (P < 0.0001; Additional file 4, Table S4) and not between aT and CON groups (P > 0.05 Additional file 4, Table S4).

**Figure 4:**
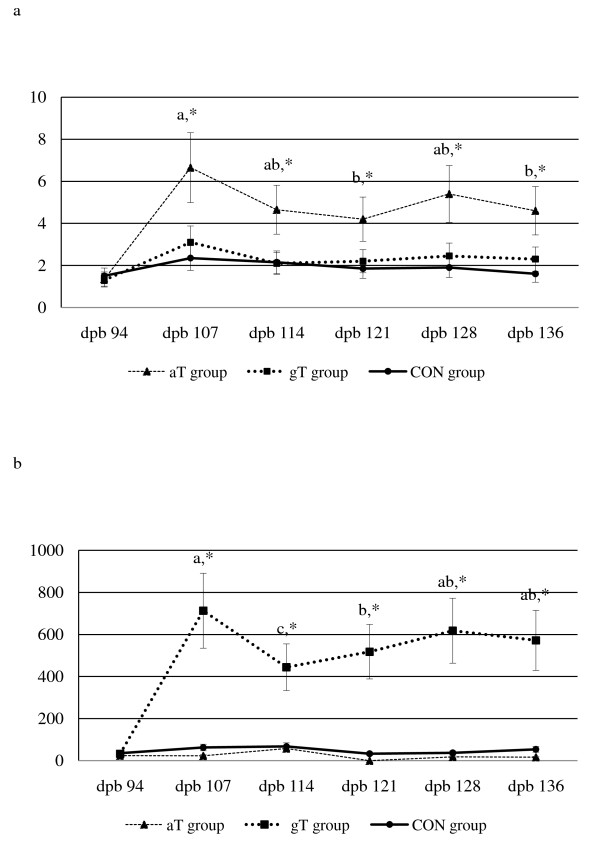
**Serum tocopherol concentrations in late pregnant ewes supplemented with alpha tocopherol, gamma tocopherol or placebo**. a. Serum alpha tocopherol concentrations (mg/kg) during late pregnancy in supplemented ewes. Line graphs represent median (25^th^,75^th ^percentile); *- aT group significantly different from gT and CON group (P < 0.001); ab - Median values with different superscripts were significantly different between days post breeding within aT group (P < 0.05); No differences between days post breeding were observed for gT and CON groups; Bar - 25^th ^and 75^th ^percentile; dpb - days post breeding; aT - alpha tocoperhol; gT - gamma tocopherol; CON - placebo; b. Serum gamma tocopherol concentration (μg/kg) during late pregnancy in supplemented ewes. Line graphs represent median (25^th^,75^th ^percentile); *- gT group significantly different from aT and CON group (P < 0.001); Median values with different letters were significantly different between days post breeding within gT group (P < 0.05); No differences between days post breeding were observed for gT and CON groups; Bar - 25^th ^and 75^th ^percentile; dpb - days post breeding; aT - alpha tocoperhol; gT - gamma tocopherol; CON - placebo.

Pair-wise comparisons of the median (25^th^, 75^th ^percentile) serum aT concentrations (excluding pre-supplementation level) were significantly higher for ewes in aT group compared to ewes in gT and CON groups during the trial period for the respective stages (P < 0.001; Figure 4a and additional file 4, Table S4). Pair-wise comparison of median tocopherol levels between stages within treatment groups was different for the aT group (P < 0.05) but not in gT and CON groups (P > 0.05). Similarly, pair-wise comparisons of the median (25^th^, 75^th ^percentile) serum gT concentrations (excluding pre-supplementation level) were significantly higher for ewes in the gT group compared to ewes in aT and CON groups during the trial period for the respective stages (Figure 4b and additional file 4, Table S4). Pair-wise comparison of median tocopherol levels between stages within treatment groups was different for the gT group (P < 0.05) but not in aT and CON groups (P > 0.1).

### Uterine and placental tocopherol concentrations

The median aT tocopherol concentration in placentome and uterus was greater in ewes supplemented with aT compared to ewes in gT and CON groups (P < 0.05; Table 2). The median gT concentrations in placentome and uterus were greater in ewes supplemented with gT compared to ewes in the aT and CON groups (P < 0.05; Table 2). The median gT in the uterus of CON ewes was greater than aT supplemented ewes (P < 0.05; Table 2).

### Vascular Endothelial Growth Factor, Placental Growth Factor, endothelial Nitrous Oxide Synthase and Hypoxia Inducible Factors expression

**Figure 5:**
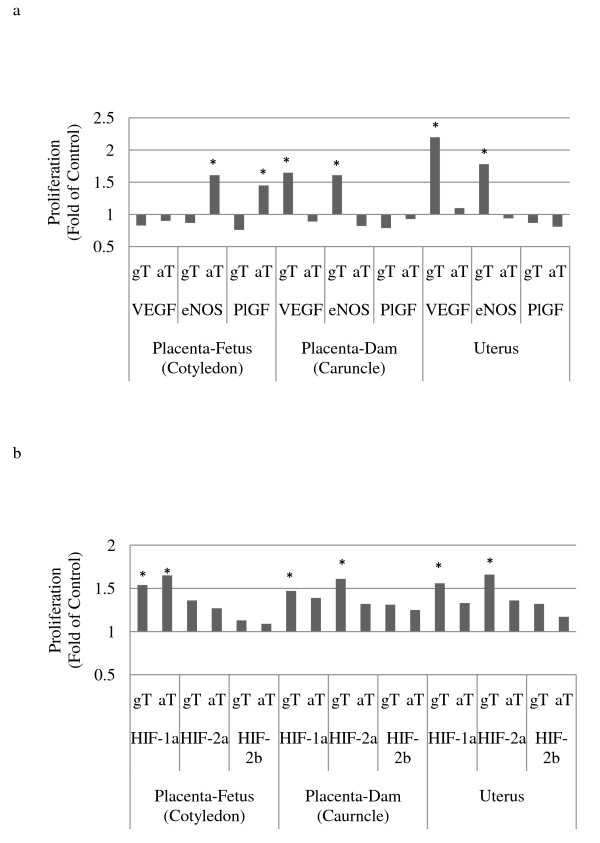
**Relative mRNA of target genes expressed in uterus and placenta of late pregnant ewes following oral supplementation of alpha tocopherol, gamma tocopherol or placebo**. a. Vascular endothelial growth factor, endothelial nitric oxide synthase, and placental growth factor mRNA expression in placenta and uterus following alpha or gamma tocopherol oral supplementation (× expression relative to control = 1; * P < 0.05); b. Hypoxia inducible factors (HIF-1α, HIF-2α, HIF-2β) expression in placenta and uterus following alpha or gamma tocopherol oral supplementation (× expression relative to control = 1; * P < 0.05).

Figure 5a shows expression of VEGF, PlGF and eNOS in placenta and uterus in tocopherol supplemented groups relative to CON, where control is 1. In cotyledon, the PlGF and eNOS expression in ewes supplemented with aT was significantly higher compared to the CON group. The VEGF expression was not different between aT and the CON groups. The VEGF, PlGF and eNOS expressions in ewes supplemented with gT were not different from the CON group. In caruncle and uterus, the VEGF and eNOS expressions in ewes supplemented with gT were significantly higher compared to the CON group. The PlGF expression was not different between gT and the CON groups. The VEGF, PlGF and eNOS expressions in ewes supplemented with aT were not different from the CON group.

The hypoxia inducible factors gene expression in tocopherol supplemented ewes relative to CON (1.0 fold) are given in Figure 5b. Ewes that received aT showed an increase in HIF-1α mRNA expression in cotyledon compared to control (P < 0.05). In gT supplemented ewes, the mRNA expressions of HIF-1α in cotyledon, and HIF-1α and HIF-2 α in caruncle and uterus were higher compared to controls (P < 0.05). There was no HIF-1β expression in cotyledon, caruncle and uterus.

### Morphometry analysis

**Figure 6:**
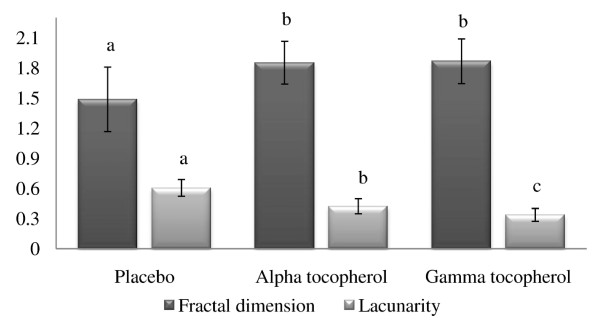
**Fractal dimension and lacunarity of placental vascular network of late pregnant ewes supplemented with alpha tocopherol, gamma tocopherol or placebo**. Bar graph represents expression of fractal dimension and lacunarity of treated groups. Groups with different letters were significant (P < 0.05).

The results showed that aT or gT supplemented ewes had increased fractal dimension and decreased lacunarity in their placental vascular network compared to CON ewes (Figure 6; P < 0.05).

## Discussion

The goal of this study was to investigate the differences in angiogenic potential of alpha- and gamma-tocopherol. We evaluated biweekly serum, and uterine and placental concentrations of tocopherols, angiogenesis gene (VEGF, PlGF, eNOS and HIFs) mRNA expressions, and morphometry of placental vascular network following daily oral supplementation in late pregnant ewes. The findings of this study indicated that oral supplementation of tocopherols to pregnant ewes during late gestation increased their serum concentrations; increased their concentrations in uterine and placentomal tissue; alpha tocopherol increased mRNA expression of PlGF, eNOS and HIF-1α in cotyledon compared to placebo; gamma tocopherol increased VEGF and eNOS mRNA expressions in caruncle and uterus, and HIF-1α in cotyledon, HIF-1α and HIF-2 α in caruncle and uterus compared to placebo; morphometry analysis of angiogenic parameters favored increased angiogenesis.

In this study, the serum and tissue level of aT (mg/kg) was higher than gT (μg/kg) even with increased gT daily dose. Gamma-tocopherol is initially absorbed in the same manner as alpha-tocopherol following administration, however, only small amounts of gamma-tocopherol are detectable in blood and tissue [32]. The bioavailability of aT is 5 to 10 times more than gT, could explain the reason for lower serum and tissue gT [33,34]. Other factors that could be responsible include high fat intake [35], oxidative stress [36,37], dose and half-life [38], species and cell type in question, and the chemical vitamer. The d2-gamma-tocopherol half-life was 13 ± 4 h compared with 57 ± 19 h for d6-alpha-tocopherol [38]. Considering the half-life, the daily oral supplementation and biweekly serum sample collection in this study occurred in the mornings, between 0800 and 0900 h, and could also be the possible explanation for the lower gamma tocopherol levels in the serum. Also, it has been demonstrated that aT transfer protein is present in early and late term placenta in other species [39]. Hence it is possible that aT was better able to transfer across to the cotyledon and thereby increased tissue aT concentration.

Tocopherols exhibit complex biological effects reflecting their diverse nutritional, antioxidant and signaling properties [26]. The biologically active range of tocopherols is relatively narrow (~3-4-fold variation). Because of this relatively narrow "window of efficacy," benefits from tocopherols might require continuous consumption to be effective. Once ingested, orally administered vitamin E plateaus in the plasma by 24 h but does not fully distribute to all biological compartments until at least ~1 week after ingestion [27]. It is consistent in this study that the serum concentrations of both aT and gT spiked during the first week and maintained at increased levels during rest of the study.

Several factors including VEGF, PlGF, basic Fibroblast Growth Factor (bFGF), epidermal growth factor, eNOS and angiopoietins 1 and 2 have been recognized as important regulators of placental development [2,40,41]. In the present study, alpha tocopherol increased mRNA expression of PlGF in cotyledon compared to placebo, and gamma tocopherol increased VEGF in caruncle and uterus. In ewes, maternal placental vascularization is dependent upon VEGF and angiopoietins expression. In contrast, fetal placental vascularization depends on numerous angiogenic factors [42,43]. It has been demonstrated that both VEGF and PlGF driven angiogenesis acts via an eNOS activated, nitric oxide mediated pathway [44]. In this study, aT increased the eNOS in cotyledon and gT increased the eNOS in caruncle and uterus. In pioneering studies, Cooney et al [14,45] showed that tocopherol reduces nitrogen dioxide to the less harmful nitric oxide. Because endothelium derived nitric oxide is a key regulator of vascular homeostasis, up-regulation of endothelial nitric oxide synthase and nitric oxide formation by tocopherol could be important in promoting angiogenesis [46].

In this study aT, increased mRNA expression of HIF-1α in cotyledon compared to placebo. The gT increased mRNA expressions of HIF-1α in cotyledon, and HIF-1α and HIF-2 α in caruncle and uterus compared to placebo. Hypoxia-inducible factor is the main regulator of the cellular response to low oxygen levels in mammalian species, playing a key role in regulating many physiological functions. The 2 principal HIF-α subunits, HIF-1α, which is ubiquitously expressed and HIF-2α, which shows a spatially restricted expression, both play important roles in vascular development and endothelial function [47-49]. Hypoxia inducible factor-1α or HIF-2α combine with HIF-1β to form the functional basic helix-loop-helix transcription factor HIF-1 to stimulate transcription of the VEGF gene via interaction with the hypoxia response element (HRE) [25]. This occurs under hypoxic conditions, but is down regulated under normoxic conditions due to targeted proteolysis of HIF-1α and HIF-2α, mediated by the von Hippel-Lindau tumor suppressor gene product (pVHL). Additionally, VEGF is regulated post-transcriptionally through stabilization of its mRNA by interaction with heterogeneous nuclear ribonuceloprotein L (hnRNP L). The basic helix-loop-helix transcription factor HIF-2α is also capable of heterodimerizing with HIF-1α to form functional HIF-1. Several genes encoding growth factors and tyrosine kinase receptors essential for endothelial cell growth and function such as EPO, VEGF, eNOS and Flk-1 were shown to contain HIF-binding sites in their promoter/enhancer sequences.

Although placental and fetal growth and development are driven by the program encoded in its genome, the genetic regulation is influenced by the intra-uterine environment in which the fetus grows. The size and nutrient transfer capacity of the placenta plays a central role in determining the prenatal growth trajectory of the fetus. Angiogenesis is the process of formation of new vessels from preexisting vessels. Although tissue hypoxia is the main drive for angiogenesis, a growing body of evidence has demonstrated that oxidative stress can also be a potent trigger for the development of new vessels. Hence it seems reasonable that the presence of no or high oxidative stress may result in formation of new vessels. Angiogenic sprouting is controlled by the balance between pro-angiogenic signals (VEGF) and factors that promote quiescence (pericyte) or VEGF inhibitors [50]. In conditions that favor angiogenesis, some endothelial cells can sprout, whereas others fail to respond. In the present study, morphometric analysis revealed ewes supplemented with alpha- or gamma-tocopherol had increased fractal dimension and decreased lacunarity compared to placebo treated ewes indicated increased angiogenesis in utero-placental vascular network. It should be noted that even though not significant reduced mRNA expressions for VEGF, PlGF and eNOS in uterus and placenta were observed in this study.

## Conclusions

This study evaluated cellular responses to supplementation in order to understand the mechanisms behind the biological effects of tocopherols on placental angiogenesis during late gestation in pregnant ewes. The tocopherol concentrations of uterus and placentome increased following oral supplementation. In aT supplemented ewes, mRNA expression of PlGF, eNOS and HIF-1α in cotyledon were greater than the CON group. In gT supplemented ewes, VEGF and eNOS mRNA expressions in caruncle and uterus, and HIF-1α in cotyledon, HIF-1α and HIF-2 α in caruncle and uterus were greater than the CON group. Morphometry analysis revealed angiogenic parameters favoring increased angiogenesis. Given the findings, it is plausible that the angiogenesis observed in this study might be a prooxidant and proangiogenesis pathway. Based on the results observed in this study we confer that the tocopherols supplementation can be employed to promote placental angiogenesis during late gestation. The oral tocopherol supplementation may be considered as an option to overcome pregnancy complications due to placental ischemia during late gestation.

## Competing interests

The authors declare that they have no competing interests.

## Authors' contributions

RK did the work of acquisition of funding, conception, design and collection of data, morphometry analysis, data analysis and interpretation, and drafting of the manuscript, tables and figures. VK contributed the design and conception, PCR analysis, collection of data and drafted the manuscript. JR contributed to the work of morphometry analyses. KDP did the work of feed supplementation, euthanasia, and collection of samples. PDS contributed to the work of postmortem tissue sample collection and histology. CDT contributed to the work of HPLC, and revised the manuscript for content and language. All authors read and approved final manuscript.

## Supplementary Material

Additional file 1 **Supplemental Table S1: Composition of hay fed free choice to the pregnant ewes during the trial period**. Click here for file

Additional file 2 **Supplemental Table S2: Average tocopherols content in supplements**. Click here for file

Additional file 3 **Supplemental Table S3: 'P' values for the effect of treatment and stage of gestation on serum concentration of alpha and gamma tocopherol in ewes (N = 18) supplemented with tocopherols**. Click here for file

Additional File 4 **Supplemental Table S4: 'P' values for the comparison of differences in the serum alpha and gamma tocopherol concentrations during different stages of gestation in ewes (N = 18) supplemented with tocopherols**. Click here for file

## References

[B1] Folkman J Klagsbrun M Angiogenic factors Science 1987 235 442 447 10.1126/science.2432664 2432664

[B2] Sherer DM Abulafia O Angiogenesis during implantation, and placental and early embryonic development Placenta 2001 22 1 13 10.1053/plac.2000.0588 11162347

[B3] Gruber M Simon MC Hypoxia-inducible factors, hypoxia, and tumor angiogenesis Curr Opin Hematol 2006 13 169 174 10.1097/01.moh.0000219663.88409.35 16567961

[B4] Teicher BA Hypoxia, tumor endothelium, and targets for therapy Adv Exp Med Biol 2005 566 31 38 full_text 16594131 10.1007/0-387-26206-7_5

[B5] Rojas A Figueroa H Re L Morales MA Oxidative stress at the vascular wall: Mechanistic and pharmacological aspects Arch Med Res 2006 37 436 448 10.1016/j.arcmed.2005.11.012 16624640

[B6] Sauer H Wartenberg M Reactive oxygen species as signaling molecules in cardiovascular differentiation of embryonic stem cells and tumor induced angiogenesis Antioxid Redox Signal 2005 7 1423 434 10.1089/ars.2005.7.1423 16356105

[B7] Liu Y Zhao H Li H Kalyanaraman B Nicolosi AC Gutterman DD Mitochondrial sources of H_2_O_2 _generation play a key role in flow-mediated dilation in human coronary resistance arteries Circ Res 2003 93 573 580 10.1161/01.RES.0000091261.19387.AE 12919951

[B8] Zafari AM Ushio-Fukai M Akers M Yin Q Shah A Harrison DG Taylor WR Griendling KK Role of NADH/NADPH oxidase derived H_2_O_2 _in angiotensin II-induced vascular hypertrophy Hypertension 1998 32 488 495 9740615 10.1161/01.hyp.32.3.488

[B9] Jones DP Radical-free biology of oxidative stress Am J Physiol Cell Physiol 2008 295 C849 868 10.1152/ajpcell.00283.2008 18684987 PMC2575825

[B10] Burton GJ Hempstock J Jauniaux E Oxygen, early embryonic metabolism and radical mediated embryopathies Reprod Biomed Online 2003 6 84 96 10.1016/S1472-6483(10)62060-3 12626148

[B11] Wiznitzer A Furman B Mazor M Reece EA The role of prostanoids in the development of diabetic embryopathy Sem Reprod Endocrinol 1999 17 175 181 10.1055/s-2007-1016224 10528368

[B12] Jauniaux E Hempstock J Greenwold N Burton GJ Trophoblastic oxidative stress in relation to temporal and regional differences in maternal placental blood flow in normal and abnormal early pregnancy Am J Pathol 2003 162 115 125 12507895 10.1016/S0002-9440(10)63803-5 PMC1851128

[B13] Jauniaux E Watson AL Hempstock J Bao Y-P Skepper JN Burton GJ Onset of placental blood flow and trophoblastic oxidative stress: a possible factor in human early pregnancy failure Am J Pathol 2000 157 2111 122 11106583 10.1016/S0002-9440(10)64849-3 PMC1885754

[B14] Traber MG Burton GW Hamilton RL Vitamin E trafficking Ann N Y Acad Sci 2004 1031 1 12 10.1196/annals.1331.001 15753129

[B15] Williamson KS Gabbita SP Mou S West M Pye QN Markesbery WR Cooney RV Grammas P Reimann-Phillip U Floyd RA Hensley K The nitration product 5-nitro-gamma-tocopherol is increased in the Alzheimer brain Nitric Oxide 2002 6 221 227 10.1006/niox.2001.0399 11890747

[B16] Cooney RV Franke AA Harwood PJ Hatch-Pigott V Custer LJ Mordan LJ γ-Tocopherol detoxification of nitrogen dioxide: Superiority to α-tocopherol Proc Natl Acad Sci USA 1993 90 1771 1775 10.1073/pnas.90.5.1771 8446589 PMC45961

[B17] Saldeen T Li D Mehta JL Differential effects of alpha- and gamma-tocopherol on low-density lipoprotein oxidation, superoxide activity, platelet aggregation and arterial thrombogenesis J Am Coll Cardiol 1999 34 1208 1215 10.1016/S0735-1097(99)00333-2 10520814

[B18] Burton GW Traber MG Vitamin E: antioxidant activity, biokinetics, and bioavailability Ann Rev of Nutr 1990 10 357 382 10.1146/annurev.nu.10.070190.002041 2200468

[B19] Ozer NK Sirikci O Taha S San T Moser U Azzi A Effect of vitamin E and probucol on dietary cholesterol-induced atherosclerosis in rabbits Free Radic Biol Med 1998 24 226 233 10.1016/S0891-5849(97)00136-6 9433896

[B20] Keaney JF Jr Gaziano JM Xu A Frei B Curran-Celentano J Shwaery GT Loscalzo J Vita JA Low-dose α-tocopherol improves and high-dose α-tocopherol worsens endothelial vasodilator function in cholesterol- fed rabbits J Clin Invest 1994 93 844 851 10.1172/JCI117039 8113416 PMC293946

[B21] Kontush A Finckh B Karten B Kohlschutter A Beisiegel U Antioxidant and prooxidant activity of α-tocopherol in human plasma and low density lipoprotein J Lipid Res 1996 37 1436 1448 8827516

[B22] Versari D Daghini E Rodriguez-Porcel M Sattler K Galili O Pilarczyk K Napoli C Lerman LO Lerman A Chronic antioxidant supplementation impairs coronary endothelial function and myocardial perfusion in normal pigs Hypertension 2006 47 475 481 10.1161/01.HYP.0000201445.77125.26 16446399

[B23] Shibata A Nakagawa K Sookwong P Tsuduki T Oikawa S Miyazawa T δ-Tocotrienol suppresses VEGF induced angiogenesis whereas r-tocopherol does not J Agri Food Chem 2009 57 8696 8704 10.1021/jf9012899 19702331

[B24] Hewitt DP Mark PJ Waddell BJ Glucocorticoids prevent the normal increase in placental vascularity during late pregnancy in the rat Endocrinology 2006 147 5568 5574 10.1210/en.2006-0825 16959835

[B25] Regnaulta TRH Galanb HL Parkera TA Anthony RV Placental development in normal and compromised pregnancies-A review Placenta 2002 23 S119 129 10.1053/plac.2002.0792 11978069

[B26] Panfili G Fratianni A Irano M Normal phase High-Performance Liquid Chromatography method for the determination of tocopherols and tocotrienols in Cereals J Agric Food Chem 2003 51 3940 3944 10.1021/jf030009v 12822927

[B27] Katsanidis E Addis PB Novel HPLC analysis of tocopherols, tocotrienols, and cholesterol in tissue Free Radic Biol Med 1999 27 1137 1140 10.1016/S0891-5849(99)00205-1 10641704

[B28] Tsatsaris V Goffin F Munaut C Brichant JF Pignon MR Noel A Schaaps JP Cabrol D Frankenne F Foidart JM Overexpression of the soluble vascular endothelial growth factor receptor in preeclamptic patients: pathophysiological consequences J Clin Endocrinol Metab 2003 88 5555 5563 10.1210/jc.2003-030528 14602804

[B29] Livak KJ Schmittgen TD Analysis of relative gene expression data using real-time quantitative PCR and the 2(-Delta Delta C(T)) Method Methods 2001 25 402 408 10.1006/meth.2001.1262 11846609

[B30] Doukas CN Maglogiannis I Chatziioannou AA Computer supported angiogensis quantification using image analysis and statistical averaging IEEE Transact Informat Tech Biomed 2008 12 650 657 10.1109/TITB.2008.926463 18779080

[B31] Talavera-Adame D Xiong Y Zhoa T Arias AE Sierra-Honigmann MR Farkas DL Quantitative and morphometric evaluation of the angiogenic effects of leptin J Biomed Optics 2008 13 064017, 1 7 10.1117/1.3028010 19123663

[B32] Traber MG Elsner A Brigelius-Flohe R Synthetic as compared with natural vitamin E is preferentially excreted as alpha-CEHC in human urine: studies using deuterated alpha-tocopheryl acetates FEBS Lett 1998 437 145 148 10.1016/S0014-5793(98)01210-1 9804189

[B33] Lodge JK Hall WL Jeanes YM Proteggente AR Physiological factors influencing vitamin E biokinetics Ann N Y Acad Sci 2004 1031 60 73 10.1196/annals.1331.006 15753134

[B34] Behrens WA Madère R Alpha- and gamma tocopherol concentrations in human serum J Am Coll Nutr 1986 5 91 106 3700885 10.1080/07315724.1986.10720116

[B35] Bates CJ Mishra GD Prentice A Gamma-tocopherol as a possible marker for nutrition-related risk: results from four National Diet and Nutrition Surveys in Britain Br J Nutr 2004 92 137 150 10.1079/BJN20041156 15230997

[B36] Ferrari L Herber R Batt AM Siest G Differential effects of human recombinant interleukin-1 beta and dexamethasone on hepatic drug metabolizing enzymes in male and female rats Biochem Pharmacol 1993 45 2269 2277 10.1016/0006-2952(93)90198-6 8517867

[B37] Shedlofsky SI Israel BC McClain CJ Hill DB Blouin RA Endotoxin administration to humans inhibits hepatic cytochrome P450-mediated drug metabolism J Clin Invest 1994 94 2209 2214 10.1172/JCI117582 7989576 PMC330046

[B38] Leonard SW Paterson E Atkinson JK Ramakrishnan R Cross CE Traber MG Studies in humans using deuterium-labeled alpha- and gamma-tocopherols demonstrate faster plasma gamma-tocopherol disappearance and greater gamma-metabolite production Free Radic Biol Med 2005 38 857 866 10.1016/j.freeradbiomed.2004.12.001 15749381

[B39] Traber MG Burton GW Hughes L Ingold KU Hidaka H Malloy M Kane J Hyams J Kayden HJ Discrimination between forms of vitamin E by humans with and without genetic abnormalities of lipoprotein metabolism J Lipid Res 1992 33 1171 182 1431596

[B40] Zygmunt M Herr F Munstedt K Lang U Liang OD Angiogenesis and vasculogenesis in pregnancy Eur J Obstet Gynecol Reprod Biol 2003 110 S10 S18 10.1016/S0301-2115(03)00168-4 12965086

[B41] Folkman J Angiogenesis in cancer, vascular, rheumatoid and other disease Nat Med 1995 1 27 31 10.1038/nm0195-27 7584949

[B42] Reynolds LP Borowicz PP Vonnahme KA Johnson ML Grazul-Bilska AT Redmer DA Caton JS Placental angiogenesis in sheep models of compromised pregnancy J Physiol 2005 15 43 58 10.1113/jphysiol.2004.081745 PMC1464490 15760944

[B43] Borowicz PP Arnold DR Johnson ML Grazul-Bilska AT Redmer DA Reynolds LP Placental growth throughout the last two thirds of pregnancy in sheep: vascular development and angiogenic factor expression Biol Reprod 2007 76 259 267 10.1095/biolreprod.106.054684 17050858

[B44] Ahmad S Hewett PW Wang P Al-Ani B Cudmore M Fujisawa T Haigh JJ le Noble F Wang L Mukhopadhyay D Ahmed A Direct evidence for endothelial vascular endothelial growth factor receptor-1 function in nitric oxide-mediated angiogenesis Circ Res 2006 99 715 722 10.1161/01.RES.0000243989.46006.b9 16946136

[B45] Cooney RV Harwood PJ Franke AA Narala K Sundstorm AK Berggren PO Mordan LJ Products of gamma-tocopherol reaction with NO2 and their formation in rat insulinoma (RINm5F) cells Free Radic Biol Med 1995 19 259 269 10.1016/0891-5849(95)00019-T 7557540

[B46] Carr A Frei B The role of natural antioxidants in preserving the biological activity of endothelium-derived nitric oxide Free Radic Biol Med 2000 28 1806 1814 10.1016/S0891-5849(00)00225-2 10946222

[B47] Semenza GL Regulation of mammalian O_2 _homeostasis by hypoxia-inducible factor 1 Annu Rev Cell Dev Biol 1999 15 551 578 10.1146/annurev.cellbio.15.1.551 10611972

[B48] Jain S Maltepe E Lu MM Simon C Bradfield CA Expression of ARNT, ARNT2, HIF1 alpha, HIF2 alpha and Ah receptor mRNAs in the developing mouse Mech Dev 1998 73 117 123 10.1016/S0925-4773(98)00038-0 9545558

[B49] Covello KL Kehler J Yu H Gordan JD Arsham AM Hu C Labosky PA Simon MC Keith B HIF-2alpha regulates Oct-4: effects of hypoxia on stem cell function, embryonic development, and tumor growth Genes Dev 2006 20 557 570 10.1101/gad.1399906 16510872 PMC1410808

[B50] Gerhardt H Golding M Fruttiger M Ruhrberg C Lundkvist A Abramsson A Jeltsch M Mitchell C Alitalo K Shima D Betsholtz C VEGF guides angiogenic sprouting utilizing endothelial tip cell filopodia J Cell Biol 2003 161 1163 1177 10.1083/jcb.200302047 12810700 PMC2172999

