# Supplementary material for: Tocopherol induced angiogenesis in placental vascular network in late pregnant ewes
Source: Reprod Biol Endocrinol. 2010 Jul 12;8:86. doi: 10.1186/1477-7827-8-86 (PMC2913989; doi:10.1186/1477-7827-8-86)
Supplement: Additional file 1 — Supplemental Table S1: Composition of hay fed free choice to the pregnant ewes during the trial period. [file 1477-7827-8-86-S1.DOC]

**Supplemental Table 1**: Composition of hay fed free choice to the pregnant ewes during the trial period

| Contents (unit) | Amount |
| --- | --- |
| Moisture | 12.7 |
| Dry matter | 87.3 |
| Crude protein (% DM) | 14.5 |
| Total Digestible Nutrients (% DM) | 53.2 |
| Acid Detergent Fiber (% DM) | 43.3 |
| Neutral Detergent Fiber (% DM) | 58.6 |
| Ash (% DM) | 7.3 |
| NFC (% DM) | 18.1 |
| Calcium (% DM) | 0.64 |
| Phosphorous (% DM) | 0.26 |
| Magnesium (% DM) | 0.30 |
| Potassium (% DM) | 1.94 |
| Sodium(% DM) | 0.039 |
| Iron (PPM) | 263 |
| Manganese (PPM) | 50 |
| Zinc (PPM) | 34 |
| Copper (PPM) | 6 |
| Alpha tocopherols (mg/kg) | 32.44 |
| Gamma tocopherols (mg/kg) | 3.62 |
| Other tocopherols (mg/kg) | 2.39 |

Hay samples were submitted to a commercial feed analysis laboratory (Cumberland Valley Analytical Services, Inc. P O Box 669, Maugansville, MD 21767) for nutrient analysis.

Method for determination of tocopherols in hay samples

The method described by Panfili et al (2003) was employed for the quantification of tocopherols in the hay samples [18]. Briefly, a 0.5 g of hay sample was placed into a 15 mm screw capped glass tube. For each sample, a dose-dependent (10, 50, 100, 500 and 1000 mg/L) external standard tocopherol (Sigma-Aldrich,
St. Louis, MO 63103) addition - as additional positive controls for HPLC-detection were prepared. The tubes were degassed with nitrogen. One mL of KOH (600g/L), 1 mL of 95% ethanol and 1 mL of NaCl_2_ (10g/L) and 2.5 mL of 1,2,3-trihydroxybenzene pyrogallic acid (pyrogallol) in ethanol (60g/L) were added and vortexed. The samples were incubated in a water-bath at 70 °C for 45 min, mixed every 10 min. After the incubation, the samples were cooled in ice and 7 mL of NaCl_2_ (10g/L) were added. The samples were extracted 3 times with 2.5 mL of cyclohexane and were centrifuged at 2240 g for 5 min to obtain a clear layer of cyclohexane. After evaporation of solvents the residues were reconstituted in 500 µL of cyclohexane and analyzed for tocopherols by HPLC.
