# Supplementary material for: Tocopherol induced angiogenesis in placental vascular network in late pregnant ewes
Source: Reprod Biol Endocrinol. 2010 Jul 12;8:86. doi: 10.1186/1477-7827-8-86 (PMC2913989; doi:10.1186/1477-7827-8-86)
Supplement: Additional file 2 — Supplemental Table S2: Average tocopherols content in supplements. [file 1477-7827-8-86-S2.DOC]

**Supplemental Table 2**: Average tocopherol content in supplements

| Product | Tocopherol Content | | |
| --- | --- | --- | --- |
|  | Alpha | Gamma | Others |
| Gamma tocopherol product | 80 mg/g | 800 mg/g | 70 mg/g |
| Alpha tocopherol product | 820 mg/g | 11 mg/g | 14 mg/g |
| Placebo | - | - | - |

**Method for determination of tocopherols in alpha and gamma tocopherol supplements**

One mL of cyclohexane containing 0.01% butylated hydroxytoluene (BHT) was added to 10 mg of supplement sample.  The samples were vortexed and were diluted 100 fold with cyclohexane containing 0.01% BHT.  The samples were filtered through a Pall Gellman Acrodisc® (0.2 µm/13mm) into a HPLC vial. The samples were analyzed by HPLC to quantify the tocopherols content.
