# Supplementary material for: Tocopherol induced angiogenesis in placental vascular network in late pregnant ewes
Source: Reprod Biol Endocrinol. 2010 Jul 12;8:86. doi: 10.1186/1477-7827-8-86 (PMC2913989; doi:10.1186/1477-7827-8-86)
Supplement: Additional file 3 — Supplemental Table S3: 'P' values for the effect of treatment and stage of gestation on serum concentration of alpha and gamma tocopherol in ewes (N = 18) supplemented with tocopherols. [file 1477-7827-8-86-S3.DOC]

**Supplemental Table 3**: ‘P’ values for the effect of treatment and stage of gestation on serum concentration of alpha and gamma tocopherol in ewes supplemented with tocopherols (N=18)

| Parameters | ‘P” value | | |
| --- | --- | --- | --- |
|  | Treatment | Gestation Stage | Treatment*Gestation Stage |
| Alpha T | 0.0001 | 0.0001 | 0.0001 |
| Gamma T | 0.0001 | 0.0001 | 0.0001 |
