# Supplementary material for: Tocopherol induced angiogenesis in placental vascular network in late pregnant ewes
Source: Reprod Biol Endocrinol. 2010 Jul 12;8:86. doi: 10.1186/1477-7827-8-86 (PMC2913989; doi:10.1186/1477-7827-8-86)
Supplement: Additional File 4 — Supplemental Table S4: 'P' values for the comparison of differences in the serum alpha and gamma tocopherol concentrations during different stages of gestation in ewes (N = 18) supplemented with tocopherols. [file 1477-7827-8-86-S4.DOC]

**Supplemental Table 4**: ‘P’ values for the comparison of differences in the serum alpha and gamma tocopherol concentrations during different stages of gestation in ewes (N=18) supplemented with tocopherols

| Tocopherol | Treatment  Groups | ‘P’ values for the differences between | | | | |
| --- | --- | --- | --- | --- | --- | --- |
|  |  | Stage 1 and 2 | Stage 1 and 3 | Stage 1 and 4 | Stage 1 and 5 | Stage 1 and 6 |
| Alpha | aT vs. gT | 0.0001 | 0.0001 | 0.0001 | 0.0001 | 0.0001 |
|  | aT vs. CON | 0.0001 | 0.0001 | 0.0001 | 0.0001 | 0.0001 |
|  | gT vs. CON | 0.08 | 0.49 | 0.54 | 0.28 | 0.12 |
| Gamma | aT vs. gT | 0.0001 | 0.0001 | 0.0001 | 0.0001 | 0.0001 |
|  | aT vs. CON | 0.13 | 0.28 | 0.40 | 0.26 | 0.16 |
|  | gT vs CON | 0.0001 | 0.0001 | 0.0001 | 0.0001 | 0.0001 |

aT – daily oral supplementation of 500 mg of alpha tocopherol;

gT – daily oral supplementation of 1000 mg of gamma tocopherol;

CON – Placebo
